# An interview with Jeffrey Okeson

**DOI:** 10.1590/2177-6709.23.6.030-039.int

**Published:** 2018

**Authors:** Jeffrey Okeson, Felipe Borges Porto, Bruno D’Aurea Furquim, Daniela Feu, Fábio Sato, Lucas Cardinal

**Affiliations:** 1 » Graduated in Dentistry at the University of Kentucky in 1972. » Professor at the University of Kentucky since 1974. » Professor, Division Chief and Director of the University of Kentucky’s Orofacial Pain Program. » Past President of the American Academy of Orofacial Pain. » Founding Diplomate of the American Board of Orofacial Pain and has serviced twice as President.; 2 » Certificate degree in Orofacial Pain and Master of Science, University of Kentucky (USA). » Certificate degree in Orthodontics and Dentofacial Orthopedics, Universidade de Pernambuco, Faculdade de Odontologia (Brazil). » Certificate degree in Orthodontics and Dentofacial Orthopedics, and Master of Dentals Science, Medical University of South Carolina (USA). » Assistant Professor of Orthodontics and Orofacial Pain, Medical University of South Carolina (USA). » Diplomate of the American Board of Orofacial Pain.; 3 » MSc in Orthodontics and Public Health Dentistry, Universidade de São Paulo, Faculdade de Odontologia de Bauru (Brazil). » PhD in Oral Rehabilitation, Universidade de São Paulo, Faculdade de Odontologia de Bauru (Brazil).; 4 » Certificate degree in Orthodontics, Universidade do Estado do Rio de Janeiro (Brazil). » Master and PhD in Orthodontics, Universidade do Estado do Rio de Janeiro (Brazil). » Professor of Orthodontics, Universidade Vila Velha (Brazil). » Associate Editor, Brazilian Journal of Health Research (RBPS). » Scientific reviewer of the American Journal of Orthodontics, The Angle Orthodontist, European Journal of Orthodontics, and Dental Press Journal of Orthodontics.; 5 » Certificate degree, Master and PhD in Oral and Maxillofacial Surgery, Universidade de Campinas, Faculdade de Odontologia de Piracicaba (Brazil). » Dentist preceptor at the Hospital dos Defeitos da Face and Hospital Geral de Vila Penteado. (Brazil).; 6 » Certificate degree in TMD and Orofacial Pain, Universidade Federal do Paraná (Brazil). » MSc in Orthodontics, Universidade Católica de Minas Gerais (Brazil) and Case Western Reserve University (USA). » PhD researcher in Dental Sciences, Universidade de São Paulo (Brazil). » Post-doc Program in Orthodontics, University of Connecticut (USA). » President Director, Deformities Orofacial Institute (Instituto DO IT) (Brazil).

**Figure:**
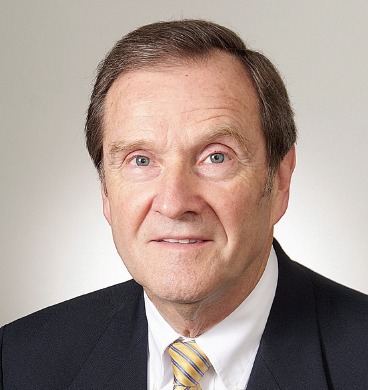

It was with great joy that I accepted the invitation from the *Dental Press Journal of Orthodontics* to coordinate an interview with Dr. Jeffrey Okeson. I have known Dr. Okeson for over 14 years. All started when I had the fantastic opportunity to be one of his residents for 3 years, and ever since, he has been my mentor and my friend. Dr. Jeffrey Okeson, despite of being a very famous and tremendously talented professional, continues to be an extremely nice and simple person. Due to these and many other reasons, I call him “my American Father”!!! He is married for 48 years to Mrs. Barbara Okeson, and they have two sons and one granddaughter. Dr. Okeson has a twin brother who is also a dentist. He enjoys jogging and has continued to jog for over 40 years. He also enjoys traveling and meeting people. Dr. Okeson has more than 240 professional publications. One of his textbooks, Management of Temporomandibular Disorders and Occlusion, is used in most of the United States dental schools and has been translated into eleven different foreign languages. Dr. Okeson has presented more than 1,200 invited lectures on the subject of TMD and orofacial pain in all 50 U.S. states and in 57 different countries. He has received multiple awards, including the first ever “Distinguished Alumni Award” from the College of Dentistry/University of Kentucky, and “The International Dentist of the Year Award” from the Academy of Dentistry International, which is the highest award recognized by this Academy. He has also been inducted into the University of Kentucky Hall of Distinguished Alumni and received the Governor’s Council on Postsecondary Education’s Acorn Award, given to top college professor in the State of Kentucky. Now is time to enjoy reading the interview!

Felipe Borges Porto (Interview coordinator)

Com grande alegria aceitei o convite do *Dental Press Journal of Orthodontics* para coordenar uma entrevista com o Dr. Jeffrey Okeson. Conheço o Dr. Okeson há mais de 14 anos. Tudo começou quando tive a fantástica oportunidade de ser um de seus residentes por 3 anos; desde então, ele tem sido meu mentor e meu amigo. Apesar de ser um profissional muito famoso e extremamente talentoso, o Dr. Jeffrey Okeson continua sendo uma pessoa extremamente simpática e simples. Devido a essas e muitas outras razões, eu o chamo de “meu pai americano”!!! Ele é casado há 48 anos com a Sra. Barbara Okeson, e eles têm dois filhos e uma neta. Dr. Okeson tem um irmão gêmeo que também é dentista. Ele adora correr, o que já faz há mais de 40 anos. Ele também gosta de viajar e conhecer pessoas. O Dr. Okeson tem mais de 240 publicações profissionais. Um de seus livros, Management of Temporomandibular Disorders and Occlusion, é utilizado na maioria das faculdades de Odontologia dos Estados Unidos, e foi traduzido para onze idiomas diferentes. Dr. Okeson apresentou mais de 1.200 palestras sobre os temas DTM e Dor Orofacial em todos os 50 estados americanos e 57 países diferentes. Ele recebeu vários prêmios, incluindo o primeiro *Distinguished Alumni Award* da College of Dentistry/University of Kentucky, e *The International Dentist of the Year Award* da Academy of Dentistry International, que é o prêmio de mais alto reconhecimento oferecido por essa academia. Ele também foi incluído no *University of Kentucky Hall of Distinguished Alumni* e recebeu o prêmio *Council on Postsecondary Education’s Acorn Award*, dado ao melhor professor universitário no estado do Kentucky. Agora, é hora de apreciar a leitura da entrevista!

Felipe Borges Porto (coordenador da entrevista)

**1) In your opinion, can occlusion have any influence on the development or aggravation of temporomandibular disorders (TMD)? (Daniela Feu)**

The role of the occlusion and TMDs has been debated for many, many years.^1^ It was initially felt that this was a cause and effect relationship. In other words, if someone had a dental malocclusion, it would cause a TMD. Likewise, if a person had a TMD problem, it would be because the occlusion was incorrect. As we progressed into evidence-based dentistry, it became obvious that this association was not nearly as consistent as we once thought. In my opinion, the evidence suggests that there are five well documented etiologic factors that are able to contribute to TMD.^1^ These factors are: occlusion, trauma, emotional stress, deep sources of pain, and parafunctional activities. These factors present as risk factors for the development of a TMD. However, there is an additional factor that needs to be considered: the adaptability of the patient. Many patients are exposed to one or more of these etiologic factors and have no evidence of TMD. It seems that many patients have significant adaptability so that less than ideal is well tolerated by them, resulting in no clinical symptoms. Therefore, in this model, occlusion may have a role in TMD, but it is only one of five potential etiologic risk factors. Yet the patient’s adaptability may override these risk factors, resulting in no TMD symptoms.

**2) What is the role (if any) of orthodontic treatment in controlling temporomandibular disorders? Are there parameters to be followed by the orthodontist during treatment and in the finalization of patient’s occlusion that may reduce the chances of developing TMD in the future? (Daniela Feu)**

**Figure 1:**
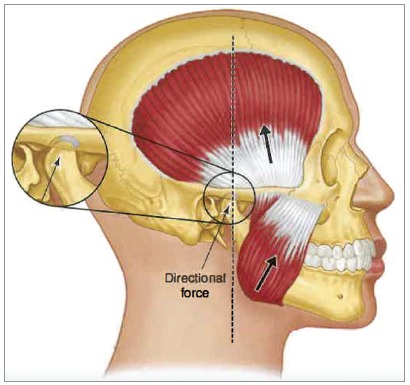
The directional forces applied to the condyles (upper thick arrow) by the temporal muscles are to seat the condyles in a superior position in the fossae. The directional forces applied to the condyles by the masseter and the medial pterygoid muscles (lower thick arrow) are to seat the condyles in a superior anterior position in the fossae (thin arrow). When these forces are combined with the lateral pterygoid muscle (not shown in the image), the condyles are seated into their superior and anterior positions in the fossae against the posterior slopes of the articular eminences. Source: Reprinted with permission from Okeson.^1^

These are very important questions. When we look at the five etiologic risk factors, orthodontic therapy can only influence one: occlusion. Therefore, from the very beginning, orthodontic therapy has only a minimum effect on TMD. However, it is important that the orthodontist recognizes how occlusion can become a risk factor for TMD. Where and how the teeth occlude has a very important relationship with temporomandibular joint function. Orthodontists should appreciate that they are truly orthopedists and need to understand the orthopedic stability in the masticatory structures. It is my belief that the most stable joint position is when the condyles are in their most superior anterior position in the fossae, resting against the posterior slope of the articular eminences, with the discs property interposed. This is an orthopedically stable joint position as determined by the muscles that load these joints. I believe it is important that in this stable joint position, the patient can achieve a stable occlusal position. The most stable occlusal position is maximum intercuspation. Therefore, orthopedic stability in the masticatory system is achieved by having both joint stability and occlusal stability that coincide. Orthodontic therapy routinely changes the patient’s occlusion. I believe every orthodontist should have as a final treatment goal to produce orthopedic stability in the masticatory structures. This means, establishing a stable joint position (Fig 1) that is in harmony with a stable occlusal position.^2^

It is reasonable to assume that orthopedic stability would reduce risk factors for developing TMD. However, producing this orthopedic stability in the masticatory structures by no means guarantees that the patient will not develop TMD. Always remember that there are four other etiologic risk factors associated with the development of TMD. I believe it is important that we do not suggest to the patient that orthodontic therapy will prevent TMD. An orthodontist may establish a stable orthopedic relationship and then other risk factors, such as trauma or emotional stress, could precipitate a TMD.

**3) The relationship between orthodontic treatment and TMD has been extensively investigated, and despite the fact that most studies indicate the lack of relationship between those, yet no consensus has been achieved. Considering the fact that all studies that investigated such relationship analyzed treatments performed by orthodontists (specialists), do you think there is a chance that poor orthodontic treatment could lead to a higher risk of developing TMD? Since there is an increase in treatments involving dental movements performed by dentist without extensive understanding in orthodontics, do you think that new longitudinal studies may start presenting stronger association between orthodontic treatment and TMD? (Felipe Porto)**

I would certainly agree that most studies investigating the relationship of orthodontic therapy and TMD do not suggest a strong association.^3^ I believe there are several reasonable explanations for these findings.

Perhaps one of these reasons is related to the discrepancy between a stable joint position and the stable occlusal position. As dentists, we have a very mechanical mind and we desire precision in our dentistry for success. Therefore, even very slight discrepancies between a stable joint position and a stable occlusal position has been described as a malocclusion. In my opinion, most patients have a discrepancy of 1-2 mm. These discrepancies have not been shown to be significantly correlated with TMD symptoms. However, in mostly epidemiological studies, slides that are greater than 3 to 4 mm from a stable joint position to a stable occlusal position become more significantly correlated to symptoms.^4^ I think the small amount of discrepancy reflects patient adaptability. It would seem reasonable that as slides become greater, the demand for adaptability becomes greater, and fewer patients have this capacity to adapt. 

Another consideration that may have influenced the results of these studies is the fact that most orthodontic therapy is accomplished in young healthy growing adolescents. Often the occlusal condition is finalized before the condyles have fully matured. When this occurs, a stable intercuspal position allows the patient to function while condyles mature into their stable functional position. Therefore, function encourages development of the condyle into its musculoskeletal stable position (form follows function). Significant discrepancies between this position and the stable occlusal position are rarely found.

Another consideration is that most, if not all, the studies that have investigated the relationship between orthodontic therapy and TMD symptoms have been accomplished in graduate orthodontic training programs. It seems reasonable to assume that treatments provided in these teaching facilities are highly supervised and at the highest level of orthodontic therapy. Perhaps orthodontic therapy that is less than ideal may pose increased risk factors, potentiating TMD symptoms. There may be some orthodontists who reviews these studies and conclude that orthodontic therapy can never influence TMD symptoms. I believe this is misinterpretation of the data and could place some patients at increased risk. I also have some concerns regarding newer orthodontic therapies that do not focus much attention on either a sound intercuspal position and/or joint position. Only time will tell if these new orthodontic technics are associated with TMD risk factors.

**4) Most of our readers are clinical orthodontists and occasionally they come across patients complaining of joint sounds that had its onset in the course of treatment. Is it possible that TMJ disc displacements are results of the orthodontic mechanotherapy, and what would you recommend to the orthodontist in these cases? (Lucas Cardinal)**

Epidemiological studies suggest that clicking is a common clinical finding in the general population. It seems to begin in the teenage years, increasing into the 20s and 30s. Studies also suggested clicking is not often associated with significant clinical symptoms.^5^ Joint sounds are common in the typical age of population undergoing orthodontic therapy. Magnuson et al^6^ have reported that clicking comes and goes between the age of 15-20, usually unrelated to significant clinical symptoms.^6^ I think it is important that the orthodontist appreciate these clinical symptoms and evaluate for them prior to beginning orthodontic treatment. Therefore, I believe it is important to conduct a history analysis and clinical examination, evaluating masticatory structures, before therapy begins. A simple screening history^1^ can be used to determine the presence of any TMD symptoms prior to treatment. Also, a simple clinical examination consisting of palpating the muscles of mastication and the TMJs, as well as observing the range of mandibular movements, is helpful to determine the presence of any TMD symptoms. These data serves as a baseline for the presence of TMD symptoms. When symptoms are present, the need for treatment should be assessed. These data are also important if, during treatment, the patient reports symptoms and believes they are a result of your treatment. Having pre-existing data is important, especially when you have documented that you discussed these findings with the patient (parents) before starting treatment.

**5) It is very common to have patients seeking orthodontic treatment because they have associated occlusal problems with TMD (muscular and/or intracapsular). In your opinion, what is the ideal treatment sequence: a) to treat the TMD first and then initiate the orthodontic treatment; b) to treat both conditions simultaneously; c) to perform orthodontic treatment monitoring the progression of the TMD? (Fábio Sato)**

As previously mentioned, I believe it is very important to assess the function of the masticatory system prior to beginning orthodontic therapy. If signs and symptoms of TMD are present, they should be discussed with the patient (parents). If the symptoms are significant, they should be managed before starting orthodontic therapy. This is important because if orthopedic instability is present, it needs to be identified as a potential risk factor for TMD symptoms. Once the symptoms have been resolved, a stable joint position can be determined so that the orthodontic therapy can provide a sound occlusal position in harmony with this joint position. A stabilization appliance may be helpful during this treatment period. 

If a stabilization appliance is utilized and the TMD symptoms are not resolved, is likely that the etiologic risk factor(s) is not malocclusion and therefore the orthodontic therapy will not likely affect the TMD symptoms. The patient should be aware of this before orthodontic therapy is begun. Achieving improved esthetics is still a possible treatment goal, but reasonable treatment expectations need to be presented to the patient prior to beginning treatment.

**6) In patients with an orthopedically unstable occlusion, severely mutilated dentition and pain, how would you approach the TMD prior to the occlusal rehabilitation? (Bruno Furquim)**

When a patient presents with TMD symptoms and also needs significant dental procedures, the TMD symptoms should be managed first, before any dental procedures are attempted (unless they are for dental infections/pain). Once the TMD symptoms have been resolved, a stable jaw position can be better identified to carry out the extensive dental procedures. Also, eliminating the TMD symptoms first may provide the clinician with insights regarding the appropriateness of the needed dental procedures.

**7) It is common for the population and for many health care professionals (including some dentists) to believe that orofacial pain is the same as TMD. Many of those professionals tend to label facial pain patients as “crazy” when TMD or dental problems are ruled out. Why do you think this happens? Where does TMD fit the full range of orofacial pain? (Felipe Porto)**

**Figure 2:**
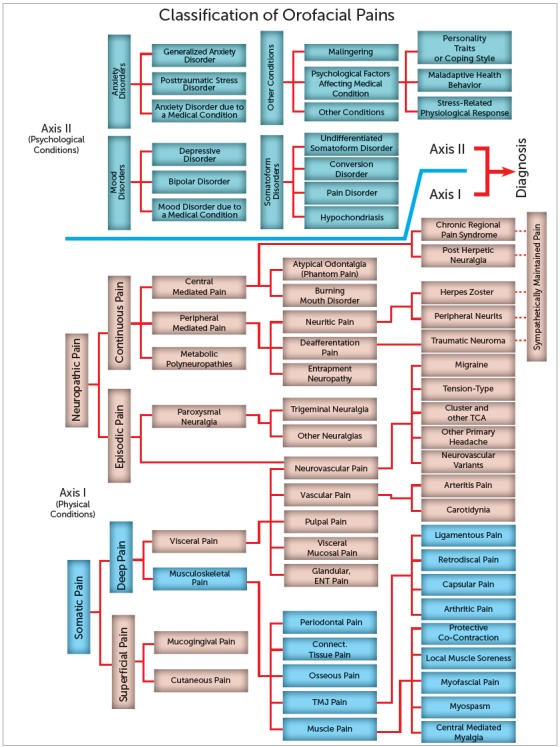
Pain classification chart, which can be used as a diagnostic “road map”. Source: Reprinted with permission from Okeson.^7^

This is a very sad, but true, statement. Many clinicians who do not see a dental cause for the pain symptoms will quickly declare that the patient is “crazy”. In my opinion, the best explanation for this is that these individuals do not have a complete understanding of orofacial pain. Temporomandibular disorders are musculoskeletal disorders of the masticatory system. There are many types of TMDs and I have a complete textbook^1^ on these disorders (Fig 2). TMDs are only one type of orofacial pain disorders and I have a second text book^7^ that reviews all of the other sources of pain in the orofacial structures. Too often clinicians focus in only what they know, and when they cannot find a reason for pain in their structures of interest, they declare the patient as “crazy”. I think part of this response is because many clinicians have a difficult time saying *“I do not know”,* and the only other alternative is to blame the patient. I have learned that telling a patient it is “all in his/her head” is extremely devastating to human spirit. In my opinion, pain in the oral and facial regions is rarely caused predominantly by psychologic factors. Certainly, pain in the face produces psychological issues such as depression, anxiety, helplessness, hopelessness and frustration. These conditions can most surely enhance the pain experience and sometimes mask other etiologies. I would hope that clinicians gain more insights to the complexity of pain and be more comfortable saying *“I do not know the reason for your pain but I will refer you to someone who may have a better understanding of what may help.”*

**8) Current literature is categorical about the high association between anxiety and temporomandibular disorders. Although association does not imply causation, we are suggested to intervene in anxiety to have better results in chronic pain management. How are these patients treated at your center? (Lucas Cardinal)**

Data certainly reveal that anxiety, and other psychosocial issues, are often associated with chronic pain conditions.^8^ One could argue whether the anxiety triggers the pain or the pain causes the anxiety. In the muscle pain categories described in my textbooks, I referred to Central Mediated Myalgia. This is a muscle pain condition that appears to have its origin more centrally associated with an upregulated autonomic nervous system.^9^ These types of pains are often frustrating for the clinician who attempts to only treat peripheral structures, such as the occlusion and/or the muscles of mastication. These treatments fail because they do not address the major contributing factor, that being an upregulated central nervous system. This pain condition becomes more prevalent as pain becomes more chronic. In our clinic, Dr. Charley Carlson, our clinical psychologist, has developed strategies by which we teach individuals how to quiet down their central nervous system.^10^ We call these behavioral skills “Physical Self-regulation Techniques” and they are well described in my TMD textbook.^1^

**9) Why do you think that many dentists believe that TMD is always related to a dental problem? And what are the implications of such belief? (Felipe Porto)**

It is very hard to break tradition. Early on, our profession assumed that TMJ disorders were predominately related to occlusion mainly because that is what we knew best. This likely relates to the old adage that states *“if the only tool you have is a hammer, the whole world starts looking like a nail”*. We were trained by mentors who we believed and trusted. However, as evidence-based medicine became more predominant in our field, we needed to review traditional belief models. As I mentioned in an earlier question, there is a potential component of occlusion in TMD, but there are many other factors that need to be considered. The clinician who has a strong belief model in occlusion will likely focus on that aspect, and not appreciate the more common etiologies related to TMD. This would not be in the best interest of the patient. There is nothing more disheartening for the patient, as for the clinician, than spending months or years changing the patient’s occlusion only to fail to resolve the pain problem. This is exactly why establishing the proper diagnosis is the most critical thing you can do for your patient. It is only after establishing the proper diagnosis that you can select the correct treatment that will be effective for the patient. In my opinion, extensive dental procedures are not necessary for most patients with TMD. However, if that is all you know, what else can you do? Do not fall into this trap. Educate yourself in this area of study so that your patients will receive the most appropriate and most conservative treatment needed to resolve the problem. 

**10) In Brazil, there is a massive and injudicious use of botulinum toxin type A in the “treatment/control” of bruxism and temporomandibular disorders in general. In your opinion, what are the indications for this drug in our specialty? (Lucas Cardinal)**

I have been using onabotulinum toxin injections in my chronic pain patients for nearly 20 years. I have learned that there are some indications, but often onabotulinum toxin injections can be misdirected and used inappropriately. It seems to have become some type of status symbol or fad, and its use needs to be placed in proper perspective. The condition I believe to be the most indicated is oromandibular dystonias. This is the most effective and appropriate treatment for these patients. However, this condition is relatively rare in a practice setting. I do not feel it is suitable for most patients with bruxism, because there is more appropriate treatment available. Onabotulinum toxin injections can certainly weaken the muscles of mastication, which reduces loading to the teeth.^11^ It does not necessarily lessen the bruxing activity. This activity is centrally driven and therefore the muscles are still active at that time, but producing less forces on the teeth. Of course, this effect last only 2-3 months and must be repeated. I think in most cases, a standard stabilization appliance can do the same thing with far less cost and greater treatment longevity. 

We are learning that onabotulinum toxin may have a positive effect on pain, far greater than just weakening muscles. Evidence is arising that onabotulinum toxin may have a positive effect on reducing neuropathic pain.^12^ ^,^ ^13^ I look forward to future investigations that may support this treatment for certain neuropathic pain conditions. In the United States, onabotulinum toxin is very expensive and not usually approved by the FDA for orofacial pain conditions. Therefore, insurance companies often do not support this treatment and it becomes very expensive for the patient. This is another factor that must be considered in treatment selection.

**11) One of the changes that I currently see in the profile of patients treated with TMD is the increasing number of children and adolescents compared to patients in the past, who were basically adults. What therapeutic approach is used in such pediatric cases, since many treatments used in adults cannot be applied in children? (Fábio Sato)**

I am not convinced the actual incidence of TMD is increasing in children and young adolescents, even though we may be seeing more of these patients. Perhaps a better explanation is that we, the professionals, have become more sensitized to these conditions and therefore identifying them earlier. I believe young adults experience similar etiologic risk factors related to TMD as do adults.^14^ I think we should generally assess and manage them using the same principles that we use with adults. The greatest difference is the presence of growth, which certainly can and should influence treatment considerations.

**12) According to your studies and experience, is there any relationship between the chronic use of mandibular advancement devices for the treatment of obstructive sleep apnea and the development of TMD? (Daniela Feu)**

**Figure 3:**
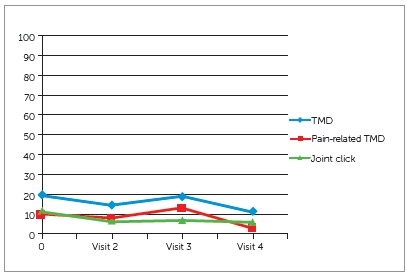
Variation of occurrence of TMD, pain-related TMD, and joint clicks on each visit. Source: Adapted with permission from Perez et al.^15^

Several years ago, one of our orofacial pain master’s students, Dr. Cristina Perez, investigated whether dental sleep appliances posed a risk factor for developing TMD (Fig 3), as well as developing a malocclusion.^15^ She recalled 167 patients who had been wearing a dental sleep appliance for between 4 to 13 months. This study reported that wearing a dental sleep appliance did not seem to increase the incidence of developing TMD. However, 18% of the patients had developed a posterior open bite. These results are now being confirmed by other studies. Dental sleep medicine clinicians are presently trying to identify the factors that led to posterior open bite so as to minimize the adverse side effect. 

**13) In your opinion, what characterize a good occlusal splint? Can it (or should it) have variations depending on specific situations? (Bruno Furquim)**

The appliance I would recommend for the most common TMD issues is a stabilization appliance. This is a hard, maxillary appliance that produces orthopedic stability in the masticatory system. This means that when the condyles are in their musculoskeletal position, the teeth occlude evenly and simultaneously, directing force through the long axis of the teeth, with adequate anterior eccentric guidance. I would only use another type of appliance for a patient who has a painful disc displacement with reduction. In that situation, I would consider a mandibular anterior positioning appliance for nighttime use only. This can help the painful retrodiscal tissue to adapt. This appliance only provides a temporary therapeutic position that would assist in adaptation, not a permanent jaw position. It should be worn nightly for 1-2 months and then eliminated once the intracapsular pain has been reduced (tissues have adapted).

Note that both these appliances should provide full arch occlusal tooth contacts. This will minimize any dental changes. The goal of these appliances is not to permanently change the patient’s occlusion; they are to help manage TMD symptoms.

**14) In recent years, I have noticed in Brazil an increase in surgeries involving the temporomandibular joint, especially the so-called minimally invasive ones, such as arthroscopy. In your opinion, what is the role of TMJ surgeries in the treatment of temporomandibular disorders? (Fábio Sato)**

There was a time in the United States when surgeries became very common for the management of TMD. We learned that routine use of surgery is not indicated or helpful in many TMD patients. The majority of TMD patients are very responsive to conservative care and more aggressive procedures are not indicated. However, there are maybe a few individuals who are relatively nonresponsive to conservative care. In these patients, a minimal surgical procedure, such as arthroscopy, may be considered. In my opinion, these procedures should only be attempted when reasonable conservative care has failed.

**15) The specialty of Temporomandibular Disorders and Orofacial Pain (TMD/OFP) in Brazil was regulated in 2002 by the Federal Council of Dentistry, but even nowadays, many professionals still do not have a complete understanding of the area of expertise of specialists in this field. Unfortunately, many dental schools in Brazil do not yet have a TMD/OFP discipline in their curriculum, and there are few specialists in the area (a little more than 1,200 in Brazil), regardless the high prevalence of this type of condition among the population. How do you see the future of the specialty for the next few years? (Fábio Sato)**

I have been in the field of TM disorders and orofacial pain for more than 40 years, and it has been very frustrating to see how slow the profession has been to recognize its importance. TMDs are the second most common pain complaint dentists face (toothache is the first). Dentists are the only healthcare providers that understand and manage TMD. Therefore, it would be appropriate for our profession to take the lead in make sure every dentist is trained in this field. This is especially true because in many cases, simple and reversible therapies are effective and easily provided. However, that is still not the case. Making this issue even more difficult is the fact that TMD is only a small part of Orofacial Pain. In United States, the Commission on Dental Accreditation has approved formal training programs in Orofacial Pain. Our program was one of the first to receive this accreditation. These are two-year, full-time programs, educating dentist in evidence-based pain concepts. It is my hope that these programs will flourish and provide sound evidence-based management for clinicians who are interested in orofacial pain. These individuals will go out into practices and be the referring specialist for the complicated chronic orofacial pain patient. It is still my belief that every dentist should have a basic understanding of TMD so that he or she can effectively manage the common, simple, transient TMD symptoms that are often managed successfully with simple, reversible therapies. However, an option should exist for clinicians who are not interested in managing TMD to send their patients to a qualified orofacial pain clinician. This is especially appropriate for the more complicated orofacial pain patient.

**16) What has changed in your way of diagnosing and treating patients with orofacial pain in the last 10 years and what do you think will change in the near future? (Lucas Cardinal)**

I believe our greatest change has been our focus away from occlusion as the major etiologic factor for TMD. Evidence-based medicine has forced us to place stronger emphasis on other risk factors, especially central mechanisms that result in orofacial pain. Perhaps the future should be directed towards a better understanding of why some patients suffer with pain more than others. A better appreciation of human adaptability would be very useful in understanding these conditions of vulnerability to pain.^16^ This has both genetic and environmental implications. Another direction of change is in the area of neuropathic pain, which are poorly understood at this time and therefore management is quite difficult. I am hopeful that better understanding of adaptation, genetics and how neurons respond to nociception may lead to more effective pain management in the future.
